# Dyslipidemia in rural areas of North China: prevalence, characteristics, and predictive value

**DOI:** 10.1186/s12944-016-0328-y

**Published:** 2016-09-13

**Authors:** Nannan Gao, Yong Yu, Bingchang Zhang, Zhongshang Yuan, Haiqing Zhang, Yongfeng Song, Meng Zhao, Jiadong Ji, Lu Liu, Chao Xu, Jiajun Zhao

**Affiliations:** 1 Department of Endocrinology, Shandong Provincial Hospital affiliated to Shandong University, Jinan, 250021 Shandong China; 2 Institute of Endocrinology and Metabolism, Shandong Academy of Clinical Medicine, Jinan, Shandong 250021 China; 3 Shandong Clinical Medical Center of Endocrinology and Metabolism, Jinan, Shandong 250021 China; 4 Department of Sonography, Provincial Hospital affiliated to Shandong University, Jinan, Shandong 250021 China; 5 Clinical Laboratory, Shandong Provincial Hospital affiliated to Shandong University, Jinan, Shandong 250021 China; 6 Department of Biostatistics, School of Public Health, Shandong University, Jinan, Shandong 250012 China

**Keywords:** Dyslipdemia, Lipoproteins, Phenotypes, Metabolic diseases, Rural China

## Abstract

**Background:**

The prevalence of cardiovascular disease has been increasing worldwide. As a common pathogenic risk factor, dyslipidemia played a great role in the incidence and progress of these diseases. We investigated to achieve accurate and up-to-date information on the prevalence of dyslipidemia and its associations with other lipid-related diseases in rural North China.

**Methods:**

Using a complex, multistage, probability sampling design, we conducted a large-scale cross-sectional study of 8528 rural participants aged over 18 years in Shandong Province. Prevalence and characteristics of dyslipidemia were demonstrated. The odds ratios between dyslipidemia types and lipid-related diseases were further analyzed by logistic regression.

**Results:**

Among the overall population, 45.8 % suffered from dyslipidemia. The prevalence of lipid abnormality (including high and very high levels) was 18.6, 12.7, 9.8 and 12.7 % for total cholesterol (TC), high-density lipoprotein (HDL), and low-density lipoprotein (LDL) cholesterol and triglycerides (TG), respectively. Among all participants with dyslipidemia, 23.9 % were aware, only 11.5 % were treated, 10.0 % were controlled. For subjects with dyslipidemia, the risk for non-alcoholic fatty liver disease (NAFLD) was highest with a 3.3-fold over that of non-dyslipidmia (OR = 3.30, *P* < 0.001); followed by hyperuricemia and diabetes mellitus (DM), while with 2-fold increase (OR = 1.99, *P* < 0.001; OR = 1.92, *P* < 0.001); with only 1.5-fold risk for atherosclerosis (AS) (OR = 1.47, *P* < 0.001). The presence of high cholesterol was mainly associated with AS, while abnormal TG was correlated with NAFLD and DM.

**Conclusions:**

Dyslipidemia has become a serious public health issue in rural North China. The rapid increase of high TC and incremental risk of high TG may contribute to the epidemic of AS, NAFLD and DM. It is imperative to develop individualized prevention and treatment guidelines according to dyslipidemia phenotypes.

**Electronic supplementary material:**

The online version of this article (doi:10.1186/s12944-016-0328-y) contains supplementary material, which is available to authorized users.

## Background

Dyslipidemia is the established modifiable risk factor for cardiovascular diseases, which account for a ubiquitous cause of morbidity and a leading contributor to mortality in most developing countries [1]. As a common pathogenic risk factor, it has been accepted that dyslipidemia accounts for the rising of diabetes mellitus (DM), atherosclerosis (AS) and non-alcoholic fatty liver disease (NAFLD) [2, 3], which all lead to the health burden worldwide.

Over the past decades, with the development of economics and changes in lifestyles, dyslipidemia has been projected to increase in absolute number in China [4–6]. Estimated by a nationally representative sample of 46,239 adults aged 20 years among Chinese population, 31.5 % or 308 million persons had borderline high or high total cholesterol, whereas 20.4 % or 196 million persons have a borderline high, high, or very high level of LDL cholesterol [6]. This study mainly paid attention on cholesterol and lacked a detailed description on triglycerides; in addition it had not provided the prevalence and characteristics of rural dyslipidemia. However, based on China’s national conditions, more than half (50.3 %) of the Chinese population are spread in rural areas [7]. Compared with the population in urban areas, people in rural areas relatively have low income, limited medical costs reimbursement and inequitable allocation of healthcare resources [8], all which pushed less health education, even lack of detailed reports for lipid profiles in contrast with urban areas.

Therefore, knowledge of the current magnitude of dyslipidemia in rural population is important for perfect health care resource allocation and prevention and management of CVD-related diseases, in turn to attenuate the society medical burden. However, in recent years, there are relatively few and timely epidemiologic descriptions that elaborating the lipid profiles and their relationships with lipid-related diseases in rural population in North China. The current study aimed (a) to depict the prevalence of dyslipidemia among rural population in 2014; (b) to estimate the awareness, treatment and control rate of dyslipidemia; (c) to determine the associations between dyslipidemia phenotypes and lipid-related diseases, in order to achieve better control of DM, AS, NAFLD and hyperuricemia among rural population.

## Methods

### Study design and groups

Participants in the cross-sectional study were obtained from rural areas of Shandong Province, using a multiple-stage stratified sampling method in 2014. A total of 12 towns were involved. Sample size in every town or village was based on geographic region, population size, and economic status [9]. All residents aged over 18 years who had been living in their current village for 5 years or longer were invited to attend the study by telephone or door-to-door visits. The participants were excluded if they were pregnant or with incomplete vital data such as age, gender, lipid profiles, serum uric acids level, blood glucose and ultrasonic examination. Finally, 8528 subjects were selected.

The study protocol was approved by the ethics committee of Shandong Provincial Hospital affiliated to Shandong University, and all participants provided written informed consent before collecting data.

### Data collection

The survey consists of health information interview, physical examination, and extensive laboratory testing. Data collection was conducted at local health stations in the participants’ residential area by well-trained medical staff. The interview included questions related to the information on demographic and social characteristics, the diagnosis and treatment of dyslipidemia and other cardiovascular events. Current smokers were defined as those who had smoked ≥100 cigarettes in their lifetime and currently smoking cigarettes at the time of the survey. Current drinking was defined as alcohol intake more than once per month during the past 12 months. Blood pressure and anthropometric measurements were measured according to standard protocols [10]. Blood pressure was presented by the means of three measurements with the participant in a sitting position after 5 min of rest using an electronic sphygmomanometer (HEM-7117; Omron, Kyoto, Japan).

Blood samples were collected from participants after an overnight fast of at least 10 h. An oral glucose tolerance test (OGTT) was given to each participant without a self-reported history of diabetes. Serum and plasma samples were shipped in dry ice. The serum lipid profiles and hepatic and renal functions were measured using an automatic biochemistry analyzer (Olympus AU5400, Tokyo, Japan) in the clinical laboratory affiliated with Shandong University. The intra-assay and interassay coefficients of variation were always below 5 % for all of the above parameters.

All participants were performed abdominal and carotid intima-media thickness (CIMT) examination. The presence of fatty liver and AS was detected by color ultrasonic diagnostic apparatus equipped with a 9 MHz linear-array transducer (Toshiba Aplio 500 Ultrasound Scanner). The subjects were in the supine position, with the head tilted backwards. Two different longitudinal-view observations of the bilateral carotid artery were made at the common carotid artery (10 mm before the bulb) and the carotid bulb (10 mm cranially to the start of the bulb), both including the left anterior wall, the left posterior wall, the right anterior wall and the right posterior wall. The radiologists sought the segments of greatest thickness and the plaque lesions along the arterial walls. The carotid artery intima-media thickness is defined as the viewable distance between the intima-lumen and the media-adventitia interface of the artery. Three values of IMT were conducted at the site of the thickest point and two adjacent points. The three measurements were averaged as the mean-IMT. The greatest value among the eight mean-IMTs was the max-IMT [11].

### Diagnostic criteria and definitions

Dyslipidemia was defined as total cholesterol (TC) ≥6.22 mmol/L (≥240 mg/dL), and/or triglycerides (TG) ≥1.70 mmol/L (≥150 mg/dL), and/or LDL cholesterol ≥4.14 mmol/L (≥160 mg/dL), and/or HDL cholesterol <1.04 mmol/L (<40 mg/dL), and/or use of lipid-lowering medications [12]. The phenotypes of dyslipidemia were defined as follows: isolated hypercholesterolemia (*Type 1*), isolated hyperglyceridemia (*Type 2*), low HDL level alone (*Type 3*) and mixed dyslipidemia, which was further defined as (a) the presence of high TC/LDL-C and high TG (*Type 4*); (b) both elevated TG levels and low HDL-C (*Type 5*) [13, 14]. A person with dyslipidemia according to the ATP III definition was considered “aware” if the participant gave a positive response to the question, “Have you ever been told by a doctor or other health professional that your blood lipid profiles level was high?” Those who reported taking lipid-lowering drugs were considered “treated”. Dyslipidemia was considered to be controlled among the population defined as having dyslipidemia if TC <6.22 mmol/L, LDL-C <4.14 mmol/L, HDL-C ≥1.04 mmol/L, and TG <1.70 mmol/L. Diabetes was defined in accordance with the American Diabetes Association 2010 criteria:(1) a self-reported previous diagnosis by health care professionals, (2) fasting plasma glucose level of 126 mg/dl (7.0 mmol/L) or higher, (3) 2-h plasma glucose level of 200 mg/dl (11.1 mmol/L) or higher, or (4) HbA1c concentration of 6.5 % or more [15]. Hyperuricemia was defined by patients with the higher level than 7 mg/dl for men, and 6 mg/dl for women [16]. Hypertension was defined as a systolic blood pressure of 140 mmHg or higher, a diastolic blood pressure of 90 mmHg or higher, or being told by a doctor or health care professional on two or more different visits that he/she had hypertension [17]. Overweight was defined as a BMI of 24.0 to 27.9, and obesity was defined as a BMI of 28.0 or higher [18]. According to current sonographic criteria: we refer to the maximum IMT (max-IMT) <0.9 mm was “normal”; the max-IMT >0.9 mm was considered indicative of thickened intima, while max-IMT >1.3 mm indicative of atherosclerotic plaque [11]. Fatty liver was diagnosed when a patient met any two of the three following ultrasonic criteria: liver and kidney echo discrepancy and presence of increased liver echogenicity (bright); unclear intrahepatic duct structure; liver far field echo decay [19]. NAFLD was excluding patients with potential cause of chronic liver disease, such as excessive alcohol consumption, hepatitis, or taking medications with a known association with fatty liver [19].

### Statistical analysis

Demographic and biochemical indexes were described in the overall population, using percentages for categorical variables and means for continuous variables. Mean levels of total, HDL, and LDL cholesterol and triglycerides were estimated for the overall population by subgroups. Differences between mean values were tested using Student’s t-test, and differences between proportions were tested using the chi-square test. Serum total, HDL, and LDL cholesterol levels were classified on the basis of the Third Report of the Expert Panel on Detection, Evaluation, and Treatment of High Blood Cholesterol in Adults [12],see supplement Additional file 1: Table S1 for specific content. The prevalence estimates of total, HDL, and LDL cholesterol categories were calculated for the overall population and by gender and age groups, and linear trends for age-specific prevalence were tested using the linear-by-linear association chi-square test. Next, we compared the awareness, treatment and control of dyslipidemia between different gender and age groups.

Logistic regression models were used to analyze the adjusted odds ratios (ORs) and 95 % confidence interval (CI) of dyslipidemia clinical types for the development of DM, NAFLD, AS and hyperuricemia, while controlling for potential confounders. The covariates in the multivariable model 2 selected for clinical importanc and for statistical significance included the age, sex, body mass index, smoking status, drinking status, liver function, hypertension, hyperuricemia, diabetes mellitus, NAFLD and atherosclerosis.

Furthermore, we analyzed the coefficient of lipid parameters for the increase of CIMT, FPG, ALT/AST and uric acids per 1-SD increase in lipid parameters. To facilitate comparisons, the levels of lipid parameters were scaled to a mean of 0 and a standard deviation (SD) of 1. Two sided *p* value less than 0.05 was considered to be significant. Statistical analyses were conducted using SPSS 18.0 for Windows (SPSS, Chicago, IL, USA).

## Results

### Basic characteristics of the overall participants

A total of 8528 (3402 men and 5126 women) participants were included in our study. The overall prevalence of dyslipidemia was 45.8 %. Social behavior characteristics and serum levels of related indexes among the participants were summarized in Table 1. Significant differences of the variables were observed, except for drinking status. Subjects with dyslipidemia were inclined to be men and had older age, higher blood pressure, BMI and waist circumference, and were more likely to have a habit of smoking currently or in the past. In addition, the levels of liver function (AST, ALT and GGT), plasma glucose, HbA1c, uric acid and eGFR were all higher in dyslipidemia patients.

**Table 1:** The basic characteristics of the study participants according to dyslipidemia status^a^

Variables	Dyslipidemia	Non-dyslipidemia	*P*-value
	(*N* = 3905)	(*N* = 4623)	
Male, %	51.4 (49.7, 53.1)	48.6 (46.9, 50.3)	<0.001
Age, ys	53.25 (52.87, 53.64)	51.05 (50.66, 51.43)	<0.001
SBP, mmHg	137.68 (137.05, 138.32)	132.08 (131.49, 132.67)	<0.001
DBP, mmHg	80.88 (80.5, 81.25)	77.61 (77.27, 77.95)	<0.001
BMI, kg/m2	26.54 (26.41, 26.67)	24.81 (24.7, 24.93)	<0.001
Waist circumferencce, cm	91.3 (90.92, 91.67)	86.04 (85.69, 86.4)	<0.001
ALT, U/L	22.25 (21.6, 22.89)	17.75 (17.39, 18.12)	<0.001
AST, U/L	24.3 (23.62, 24.98)	22.8 (22.51, 23.09)	<0.001
GGT,U/L	37.71 (36.46, 38.95)	24.53 (23.7, 25.37)	<0.001
Creatinine	68.97 (68.51, 69.44)	64.87 (64.47, 65.26)	<0.001
Uric acid, umol/L	338.22 (335.38, 341.06)	298.52 (296.19, 300.85)	<0.001
Fasting glucose, mmol/L	6.51 (6.44, 6.58)	5.82 (5.77, 5.86)	<0.001
2-h postload glucose, mmol/L	9.23 (9.06, 9.4)	7.82 (7.7, 7.93)	<0.001
HbA1c	6.33 (6.27, 6.38)	5.92 (5.88, 5.95)	<0.001
Smoking state, %			<0.001
never	43.6 (42.4, 44.9)	56.4 (55.1, 57.6)	
former	51.9 (48.2, 55.6)	48.1 (44.4, 51.8)	
current	52.1 (49.5, 54.6)	47.9 (45.4, 50.5)	
Drinking state, %			0.105
never	44.9 (43.6, 46.3)	55.1 (53.7, 56.4)	
former	46.5 (42.0, 50.9)	53.5 (49.1, 58.0)	
current	47.4 (45.5, 49.3)	52.6 (50.7, 54.5)	

^a^Mean values (95 % confidence interval) or percentages (95 % confidence interval) are shown. *SBP* systolic blood preesure, *DBP* dystolic blood preesure, *BMI* body mass index

### Prevalence of dyslipidemia among the overall population according to age and gender

The mean levels of lipid profiles among the overall population (*N* = 8028) were shown in Additional file 1: Table S2 by age, gender, BMI and fasting plasma glucose (FPG) groups.

**Fig. 1:**
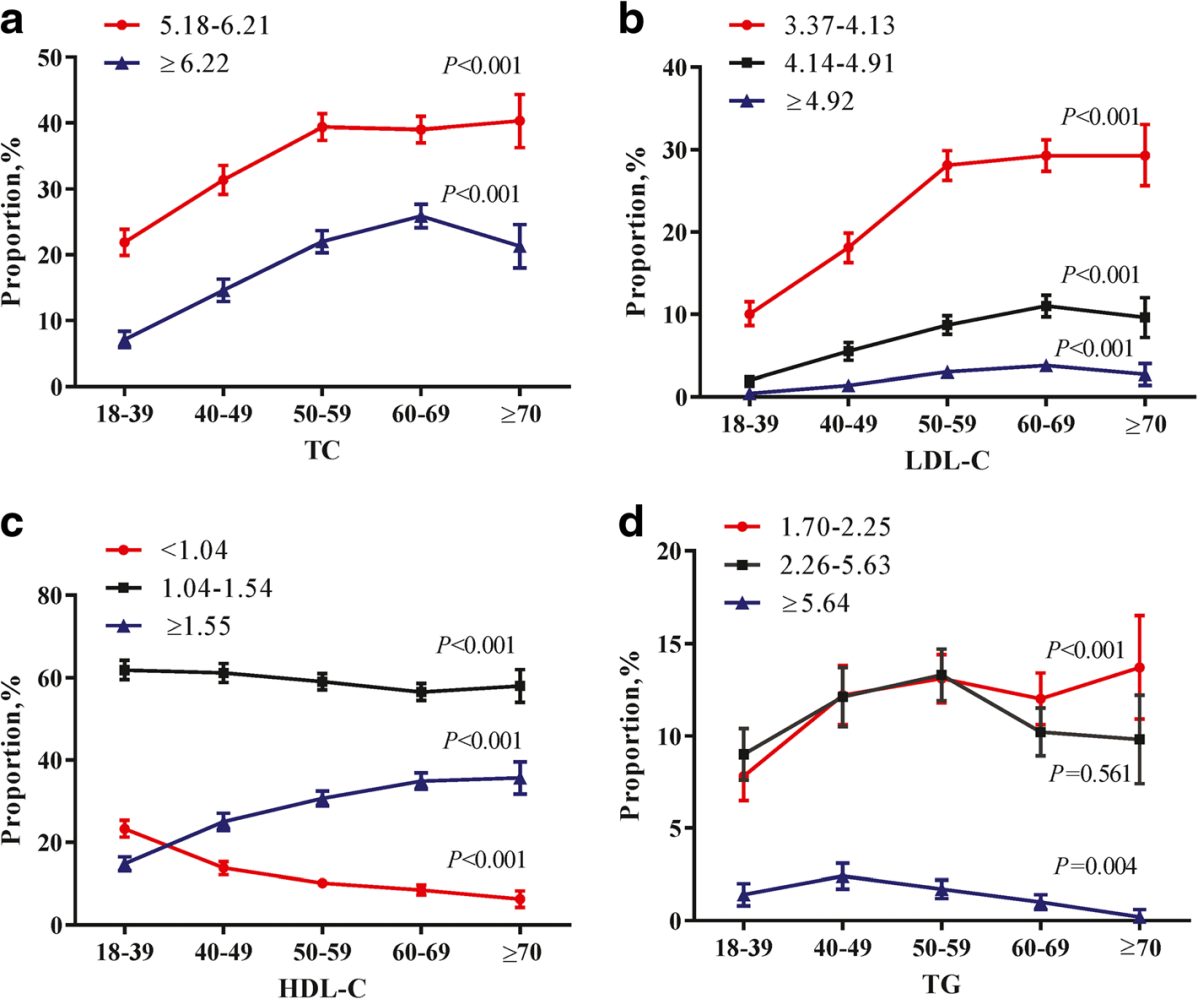
Trends in age-specific proportion (95 % Confidence Interval) of ATP III classification of total (**a**), LDL (**b**), and HDL (**c**) cholesterol and triglycerides (**d**) in the overall participants. Linear trends were tested using linear-by-linear association

Table 2 and Fig. 1 depicted the proportions of adults with lipid fractions by gender and age. Lipid profiles were categorized according to ATP III classifications. As is shown in Table 2, half (53 %) of all participants had abnormal levels of total cholesterol, and among them, high TC (≥6.22 mmol/L) accounted for one-third. While only 32.8 % of the overall population had increased LDL-cholesterol levels, mainly with borderline high level (3.37–4.13 mmol/L). Additionally, nearly one fourth suffered from TG abnormality, and among these, borderline high (1.70–2.25 mmol/L) and high (2.26–5.63 mmol/L) concentrations had the most cases; very high TG (≥5.64 mmol/L) was less than 2 %. Prevalence of low HDL cholesterol was 12.7 % for the overall population. Men were inclined to suffer higher prevalence of lipid abnormities than women (Table 2).

**Table 2:** Gender-specific proportion (95 % Confidence Interval) of ATP III classification of total, LDL, and HDL cholesterol and triglycerides in the overall participants

	Prevalence		
	Overall	Men	Women
Total cholesterol, mmol/L			
<5.18	47.0 (45.9, 48.1)	46.3 (44.6, 48.0)	47.5 (46.1, 48.9)
5.18–6.21	34.4 (33.4, 35.4)	35.6 (34.0, 37.2)	33.6 (32.3, 34.9)
≥6.22	18.6 (17.8, 19.4)	18.2 (16.9, 19.5)	18.9 (17.8, 20.0)
LDL cholesterol, mmol/L			
<2.59	29.1 (28.1, 30.1)	26.5 (25.0, 28.0)	30.9 (29.6, 32.2)
2.59–3.36	38.0 (37.0, 39.0)	39.0 (37.4, 40.6)	37.4 (36.1, 38.7)
3.37–4.13	23.0 (22.1, 23.9)	25.2 (23.7, 26.7)	21.6 (20.5, 22.7)
4.14–4.91	7.4 (6.8, 8.0)	7.1 (6.2, 8.0)	7.6 (6.9, 8.3)
≥4.92	2.4 (2.1, 2.7)	2.2 (1.7, 2.7)	2.5 (2.1, 2.9)
HDL cholesterol, mmol/L			
<1.04	12.7 (12.0, 13.4)	19.5 (18.2, 20.8)	8.3 (7.5, 9.1)
1.04–1.54	59.3 (58.3, 60.3)	58.7 (57.0, 60.4)	59.8 (58.5, 61.1)
≥1.55	27.9 (26.9, 28.9)	21.9 (20.5, 23.3)	32.0 (30.7, 33.3)
Triglycerides, mmol/L			
<1.70	75.7 (74.8, 76.6)	71.4 (69.9, 72.9)	78.5 (77.4, 79.6)
1.70–2.25	11.6 (10.9, 12.3)	12.0 (10.9, 13.1)	11.4 (10.5, 12.3)
2.26–5.63	11.2 (10.5, 11.9)	13.8 (12.6, 15.0)	9.5 (8.7, 10.3)
≥5.64	1.5 (1.2, 1.8)	2.8 (2.2, 3.4)	0.6 (0.4, 0.8)

*LDL* low density lipoprotein, *HDL* high density lipoprotein

Trends in age-specific prevalence of lipid abnormities were presented in Fig. 1. With aging, proportions for abnormal TC and LDL-C increased linearly (*P* < 0.001), and reached a peak in 60–69 group, then stayed flat or declined slightly (Fig. 1a, b). Borderline high TG concentrations (1.70–2.25 mmol/L) were basically linear increase, summit in 70 years and older (*P* < 0.001, Fig. 1d). However, prevalence of very high TG level declined with advanced age (*P* = 0.004, Fig. 1d). Lower levels of HDL-C may increase the cardiovascular risk. As the figure shown, proportions of this low HDL (<1.04 mmol/L) concentrations decreased with increased age group (*P* < 0.001, Fig. 1c).

### Awareness, treatment and control of dyslipidemia

**Fig. 2:**
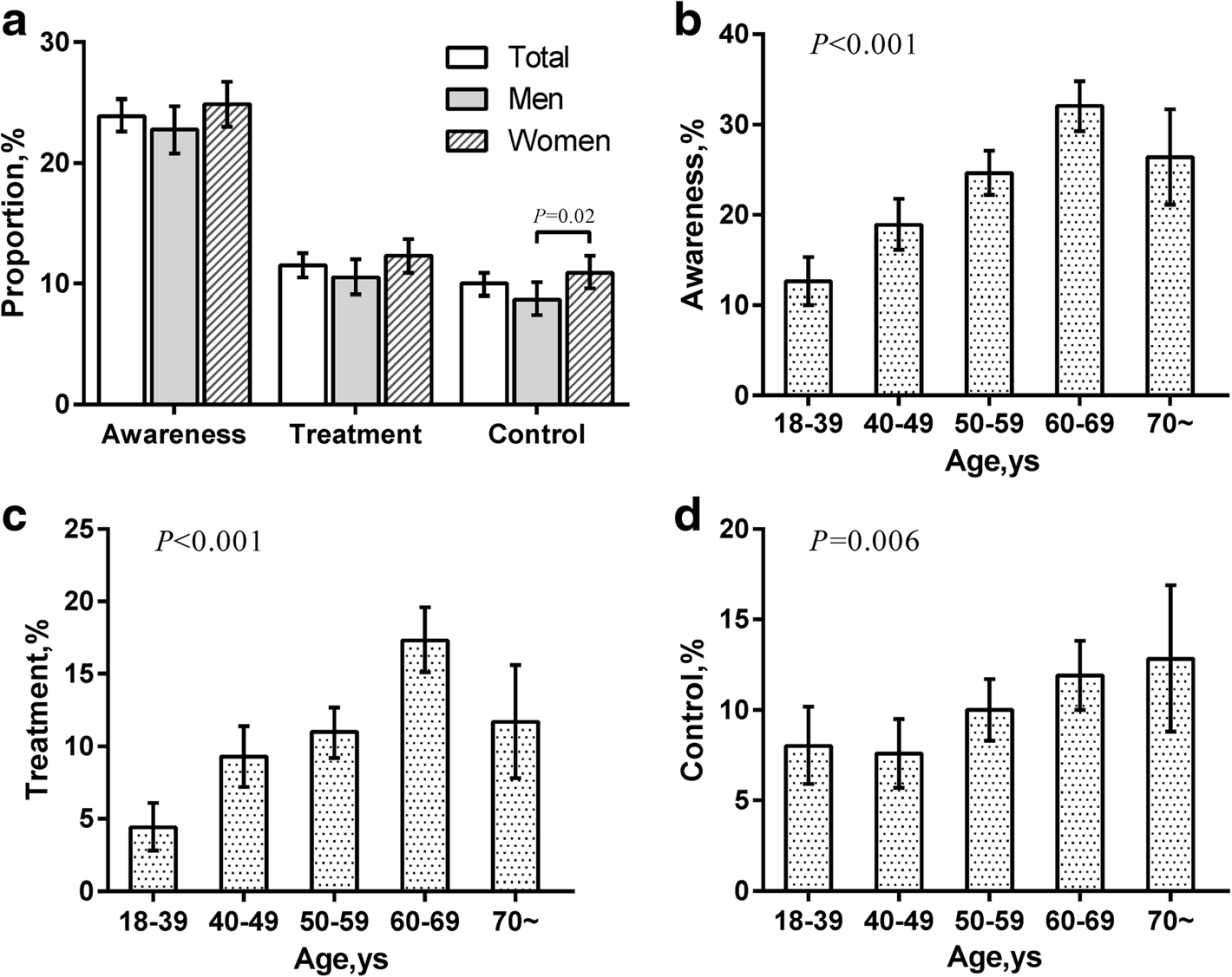
Awareness, treatment and control of dyslipidemia. **a** The total and gender-specific awareness, treatment and control of dyslipidemia. **b** The age-specific awareness of dyslipidemia. **c** The age-specific treatment of dyslipidemia. **d** The age-specific control rate of dyslipidemia.*P <0.05

Subsequently, Fig. 2 depicted the age- and gender-specific awareness, treatment and control of dyslipidemia. Among all participants with dyslipidemia, 23.9 % (22.6 %, 25.3 %) were aware, only 11.5 % (10.5 %, 12.5 %) were treated, 10.0 % (9.0 %, 10.9 %) were controlled. Compared with men, women were more likely to be aware and treated, though not statistically significant. Moreover, women have a significantly higher control rate (*P* = 0.02, Fig. 2a). The proportion of awareness, treatment for dyslipidemia significantly increased with age until peaks for 60–69 age group (Fig. 2b, c). Despite of increase over age ranges as well, the control rates hit the peak in 70 years and older (Fig. 2d).

### Associations between clinical types of dyslipidemia and lipid-related diseases

The prevalence of dyslipidemia type among the overall population was depicted in the Additional file 1: Table S3. In our results, mixed dyslipidemia (13.8 %) was the most common pattern, isolated hypercholesterolemia (*Type 1*,11.0 %) and isolated hypertriglyceridemia (*Type 2*, 10.6 %) came second. Prevalence of low HDL-C level (*Type 3*, 5.9 %) was the lowest (Additional file 1: Table S3). Among subjects with mixed dyslipidemia, 57.9 % (*N* = 681) were presence of high TC/LDL-C combined with high TG *(Type 4)*; 41.8 % (*N* = 491) were the combination of high TG with low HDL-C level (*Type 5*) (Data are not shown).

**Fig. 3:**
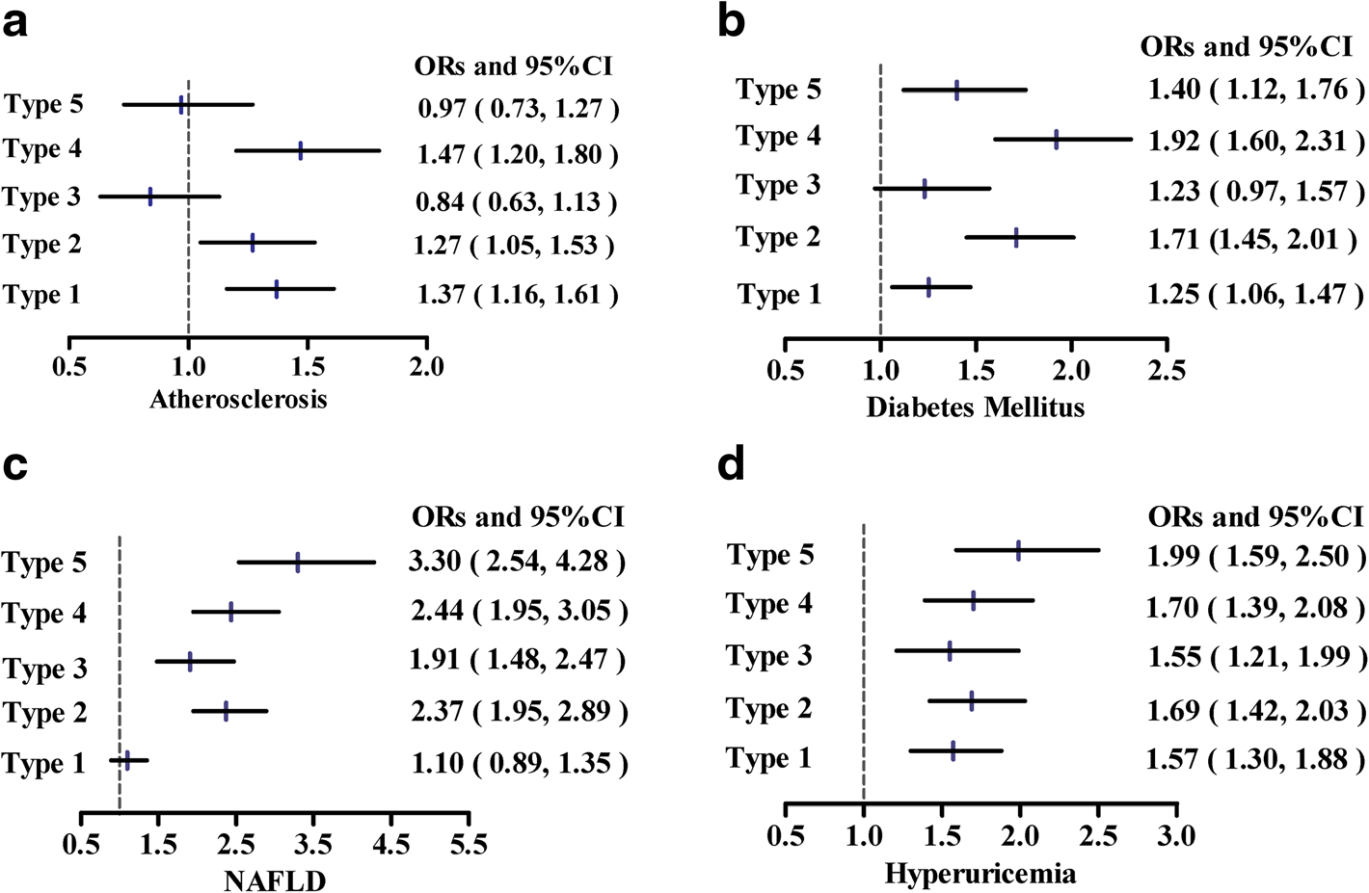
Adjusted odds ratios for the development of atherosclerosis (**a**), diabetes mellitus (**b**), NAFLD (**c**) and hyperuricemia (**d**) when associated with different lipid combinations, compared with the subgroup without dyslipidemia. Type 1, isolated hypercholesterolemia; Type 2, isolated hypertriglyceridemia; Type 3, low HDL-C level alone; Type 4, high TC/LDL-C + high TG;Type 5, high TG + low HDL-C

We then estimated the risks for development of AS, DM, NAFLD and hyperuricemia. Figure 3 showed the ORs and 95 % CI for the above five dyslipidemia types when adjusted potential confounding factors. Patients with *type 4* had the highest risk for development of AS, nearly 1.5 times over that of non-dyslipidemia (Fig. 3a, OR = 1.47, *P* < 0.001). For *type 1* and *type 2*, the risks of AS were increased by 1.4 and 1.3 fold, respectively (Fig. 3a, OR = 1.37, *P* < 0.001; OR = 1.27, *P* < 0.001). Analogously, in comparison with other phenotypes, patients with *type 4* were also most likely to suffer from diabetes, and risk had almost increased 200 % than non-dyslipidemia (Fig. 3b, OR = 1.92, *P* < 0.001); *type 2* came subsequently.

When taking NAFLD into our logistics analysis, patients with *type 5* had the greatest risk of NAFLD than other types, a 3.3-fold increase in risk was found (Fig. 3c, OR = 3.30, *P* < 0.001). *Type 1* was not significant for NAFLD (Fig. 3c, *P* = 0.396). We conducted similar analysis for the development of hyperuricemia, the ORs of above types were all significant, with *type 5* highest (Fig. 3d, OR = 1.99, *P* < 0.001).

As was shown in Additional file 1: Table S3, we further evaluated the concentration-effect relationships between the significantly single lipid parameter and the objective indexes for AS, DM, NAFLD and hyperuricemia. For example, TC/LDL-C had a positive association with CIMT, and in other words, the CIMT thickness were elevated by 0.038/0.048 respectively as every increase in the SD of the TC and LDL-C levels. By such analogy, positive correlations were observed between the level of TC/LDL-C/TG and fasting glucose. Additionally, TG had positive relation with ALT or AST, for HDL, the correlation was reverse with ALT only. And, positive correlations were found between TC, LDL-C, TG and serum uric acids, reversely for HDL-C (Additional file 1: Table S4).

## Discussion

In this study, nearly half (45.8 %) participants aged 18 years suffered dyslipidemia from rural areas in Shandong Province, with low awareness, treatment and control. Logistic regression indicated that the presence of high cholesterol was mainly associated with AS. And triglyceride abnormality was closely associated with the development of NAFLD and DM.

Our results suggested that the prevalence of dyslipidemia had increased by 2.5 folds than that in 2002 (18.6 %) among the Chinese population [5]. Furthermore, the lipid profiles have changed greatly. Combined previous rural study [20], the prevalence of high TC had raised by 7-8 folds than the proportion reported in 2002 [5]. Compared to the results of some urban areas such as Beijing and Shenzhen, the values of HDL-C and TG were also gradually approaching the urban levels, for f TC, the level was almost higher [21]. Prevalence of western diets (high intake of fat and cholesterol) in current China contributes largely to such phenomenon [22]. These striking findings alarmed the rapid epidemic trend of dyslipidemia in rural China, which have emerged as importantly modifiable risk factors in predicting CVD morbidity and mortality, together with obesity and smoking [23].

A meta-analysis study previously reported that awareness, treatment, and control rates of dyslipidemia in China were 24.4 % (95 % CI: 14.4–38.4 %), 8.8 % (95 % CI: 7.7–10.0 %), and 4.3 % (95 % CI: 4.1–4.5 %) [24], which were in accordance with our results. The high prevalence and poor control called for urgent attention on dyslipidemia than ever before in China, which undoubtedly will depress the subsequent morbidity and mortality, raise economic benefit and save hygienic resources for the whole society in the long run. It is worth mentioning that some developed countries had noticed the fact and achieved desirable succeeds. For example, in recent years, favorable trends in lipid levels have occurred in Americans, with the awareness of dyslipidemia on CVD risks [25]. The mean levels of TC declined from 5.44 to 5.18 mmol/L; between 1976 to 1980 and 1999 to 2006. Additionally, the mean concentrations of LDL-C declined from 3.37 to 3.08 mmol/L during 1976–1980 and 1999–2006.

Although the mechanisms between abnormal lipids and lipid-related diseases had been partly tested, there are conflicting data about the role of single lipid changes and certain lipid combination on other concurrent diseases. Notably, the prevalence of dyslipidemia now has increased greatly and lipid profiles have changed apparently. In that way, were the above changes associated with other non-communicable diseases? Therefore, we further explored the odd ratios between the above lipid phenotypes and AS, DM, NAFLD and hyperuricemia, which is beneficial for health-workers to establish the orientation and emphasis of prevention and treatment in the future.

Our results showed that the risks of dyslipidemia have altered obviously. The findings in the Multi-Ethnic Study of Atherosclerosis (MESA) previously also found combined hyperlipidemia and simple hypercholesterolemia were associated with increased CIMT [26], which was congruent with our logistic regression results. In these lipid combinations, high cholesterol was general, illustrating the important effect on the development on AS. Combined with the high prevalence of abnormal high TC, these findings further provided strong evidence for the higher incidence of CVD risks; moreover they strengthened the rationale for the priority of circulating cholesterol concentrations over both HDL-C and TG levels as the primary target for lipid therapy in ATP III program [12].

For patients with dyslipidemia, the risk for NAFLD was highest with a 3.3-fold over that of non-dyslipidemia; followed by hyperuricemia and diabetes, with 2-fold increase. These results may be an explanation for the striking increase of prevalence of NAFLD and DM [27–29]. In this study, the common dyslipidemia type for patients with NAFLD and DM was *type 4*, *type 5* and *type 2*, and the core lipid component was the presence of high TG. These findings implied the great risk of TG in the development of DM and NAFLD, which had common lipid-induced pathogenesis-lipotoxicity. Previous studies had shown that diabetes and NAFLD were both characterized by elevated triglycerides, low HDL-cholesterol and the predominance of small dense LDL particles [29–32]. The accumulation of TGs in liver and pancreas undoubtedly result in the disturbance of lipids balance and overproductions of free fatty acids, which would trigger the insulin resistance and mitochondrial dysfunction attributable for the damage of liver and islet normal function [31, 33]. Compared with high cholesterol, the abnormality of TG was more closely associated with diet and lifestyle changes, more easy and feasible to modify, therefore it is easier to achieve control and benefit quickly for patients, which also may reduce the risk of NAFLD and DM in the long run.

Certain potential limitations may exist in our study. First, women were overrepresented due to large number of male migrant workers ineligible for this investigation, and the results may be not suitable for other ethnicity. Secondly, this was a cross-sectional study, and therefore any causality cannot be proved.

## Conclusion

In summary, dyslipidemia has become a serious public health problem in rural population of North China. There is lack of awareness, treatment and effective control among the population. The rapid increase of high TC and higher risk of high TG may give rise to the high epidemic of AS, NAFLD and DM. It is urgent and imperative to appropriately develop individualized prevention and treatment guidelines within one person according to the dyslipidemia type, which undoubtedly depress the subsequent morbidity and mortality, raise economic benefit and save hygienic resources for the whole society.

## Additional file

Additional file 1: Table S1. ATP III classification of total, LDL, HDL cholesterol and triglycerides (mmol/L). **Table S2.** Mean (95 % Confidence Interval) of serum total cholesterol, HDL cholesterol, LDL cholesterol, and triglyceride levels in the overall participants. **Table S3.** The proportions (95 % Confidence Interval) of four dyslipidemia phenotypes according to gender, age, BMI and fasting glucose. **Table S4.** Multivariable Analyses of the Risk for Development of Lipid-related Diseases in Subjects with Different Dyslipidemia Phenotypes. (PDF 88 kb)
