# Supplementary material for: Dyslipidemia in rural areas of North China: prevalence, characteristics, and predictive value
Source: Lipids Health Dis. 2016 Sep 13;15(1):154. doi: 10.1186/s12944-016-0328-y (PMC5020547; doi:10.1186/s12944-016-0328-y)
Supplement: Additional file 1: Table S1. — ATP III classification of total, LDL, HDL cholesterol and triglycerides (mmol/L). Table S2. Mean (95 % Confidence Interval) of serum total cholesterol, HDL cholesterol, LDL cholesterol, and triglyceride levels in the overall participants. Table S3. The proportions (95 % Confidence Interval) of four dyslipidemia phenotypes according to gender, age, BMI and fasting glucose. Table S4. Multivariable Analyses of the Risk for Development of Lipid-related Diseases in Subjects with Different Dyslipidemia Phenotypes. (PDF 88 kb) [file 12944_2016_328_MOESM1_ESM.pdf]

SUPPLEMENTAL MATERIAL

Table S1. ATP III classification of total, LDL, HDL cholesterol and triglycerides (mmol/L).

Total cholesterol		HDL cholesterol	
< 5.18	Desirable	<1.04	Low
5.18-6.21	Borderline high	1.04-1.54	Borderline high
≥6.22	High	≥1.55	High
LDL cholesterol		Triglycerides	
< 2.59	Optimal	<1.70	Normal
2.59-3.36	Near or above optimal	1.70-2.25	Borderline high
3.37-4.13	Borderline high	2.26-5.63	High
4.14-4.91	High	≥5.64	Very high
≥4.92	Very high		

LDL, low density lipoprotein; HDL, high density lipoprotein.

Table S2. Mean (95% Confidence Interval) of serum total cholesterol, HDL cholesterol, LDL cholesterol,and triglyceride levels in the overall participants.

	Total cholesterol	LDL cholesterol	HDL cholesterol	Triglycerides
Total	5.34 (5.32,5.36)	3.04 (3.02,3.06)	1.38 (1.37,1.39)	1.48 (1.45,1.51)
Gender				
male	5.35 (5.31,5.39)	3.08 (3.05,3.11)	1.32 (1.30,1.33)	1.68 (1.63,1.74)
female	5.33 (5.30,5.36)	3.01 (2.98,3.03)	1.42 (1.42,1.43)	1.34 (1.32,1.37)
P value	0.374	<0.001	<0.001	<0.001
Age,ys				
18-39	4.77 (4.72,4.82)	2.51 (2.47,2.54)	1.24 (1.23,1.26)	1.32 (1.25,1.38)
40-49	5.21 (5.16,5.25)	2.91 (2.88,2.95)	1.35 (1.33,1.36)	1.59 (1.51,1.68)
50-59	5.53 (5.48,5.57)	3.21 (3.18,3.24)	1.41 (1.4,1.43)	1.58 (1.52,1.63)
60-69	5.61 (5.57,5.66)	3.30 (3.27,3.34)	1.45 (1.44,1.47)	1.43 (1.39,1.48)
≥70	5.5 (5.42,5.59)	3.20 (3.13,3.27)	1.46 (1.43,1.48)	1.36 (1.30,1.43)
P value	<0.001	<0.001	<0.001	<0.001
FPG, mmol/L				
<5.6	5.14 (5.1,5.17)	2.86 (2.83,2.88)	1.39 (1.38,1.40)	1.27 (1.24,1.31)
5.6-6.9	5.48 (5.44,5.51)	3.18 (3.15,3.21)	1.39 (1.38,1.40)	1.54 (1.49,1.58)
≥7.0	5.68 (5.62,5.75)	3.31 (3.26,3.36)	1.34 (1.32,1.35)	2.03 (1.92,2.14)
P value	<0.001	<0.001	<0.001	<0.001
BMI, kg/m2				
≤23.9	5.22 (5.18,5.26)	2.88 (2.85,2.91)	1.51 (1.50,1.52)	1.11 (1.08,1.14)
24.0-27.9	5.38 (5.35,5.41)	3.10 (3.07,3.13)	1.34 (1.33,1.35)	1.56 (1.52,1.61)
≥28	5.43 (5.39,5.48)	3.16 (3.13,3.20)	1.26 (1.25,1.27)	1.86 (1.79,1.93)
P value	<0.001	<0.001	<0.001	<0.001

FPG, fasting plasma glucose; BMI, kg/m2; LDL, low density lipoprotein; HDL, high density lipoprotein.

Table S3. The proportions (95% Confidence Interval) of four dyslipidemia phenotypes according to gender, age, BMI and fasting glucose.

	Isolated hypercholesterolemia	Isolated hypertriglyceridemia	Mixed dyslipidemia	Low HDL level alone
Total	11.0 (10.3,11.7)	10.6 (9.9,11.2)	13.8 (13.1,14.5)	5.9 (5.4,6.3)
Gender				
male	9.6 (8.6,10.6)	10.9 (9.8,11.9)	17.8 (16.6,19.1)	8.6 (7.7,9.6)
female	11.9 (11.1,12.8)	10.4 (9.6,11.2)	11.1 (10.2,12.0)	4 (3.5,4.6)
P value	0.001	0.482	<0.001	<0.001
Age,ys				
18-39	3.6 (2.7,4.5)	5.5 (4.4,6.6)	12.8 (11.1,14.4)	12.3 (10.7,13.9)
40-49	7.4 (6.1,8.6)	13.1 (11.5,14.7)	13.7 (12.0,15.3)	6.1 (5,7.3)
50-59	12.4 (11.1,13.7)	12.9 (11.5,14.2)	15.2 (13.7,16.6)	4 (3.2,4.7)
60-69	17.2 (15.7,18.8)	9.8 (8.5,11.0)	13.6 (12.1,15.0)	3.7 (2.9,4.5)
≥70	13 (10.3,15.8)	11.5 (8.9,14.1)	12.2 (9.5,14.8)	2.9 (1.6,4.3)
P value	<0.001	<0.001	0.148	<0.001
FPG,mmol/l				
<5.6	9.2 (8.3,10.1)	8 (7.2,8.8)	9.6 (8.7,10.5)	7 (6.2,7.7)
5.6-6.9	12.7 (11.5,13.8)	11.9 (10.7,13)	15.1 (13.8,16.4)	5 (4.2,5.8)
≥7.0	13.2 (11.3,15)	16.2 (14.2,18.3)	24.5 (22.1,26.9)	4.3 (3.1,5.4)
P value	<0.001	<0.001	<0.001	<0.001
BMI, kg/m2				
≤23.9	12.9 (11.7,14.2)	5.4 (4.6,6.2)	6.0 (5.1,6.8)	3.8 (3.1,4.5)
24.0-27.9	10.3 (9.3,11.3)	11.9 (10.8,13.0)	15.3 (14.1,16.5)	6.1 (5.3,6.9)
≥28	9.4 (8.1,10.6)	15.7 (14.1,17.2)	22.3 (20.5,24.1)	8.3 (7.1,9.5)
P value	0.001	<0.001	<0.001	<0.001

FPG, fasting plasma glucose; BMI, kg/m2; LDL, low density lipoprotein; HDL, high density lipoprotein. Isolated hypertriglyceridemia: TG≥1.7mmol/L and TC<6.22 mmol/L and LDL-C<4.14mmol/L and HDL>1.04mmol/L; isolated hypercholesterolemia: TC≥6.2/LDL-C≥4.14 and triglycerides<1.7 mmol/L and HDL>1.04mmol/L; mixed hyperlipidemia: triglycerides≥1.7 mmol/L and TC≥6.2 mmol/L/ LDL-C≥4.14mmol/L; isolated low HDL-C: HDL-C≤1.04 mmol/L without hypertriglyceridemia nor hypercholesterolemia.

Table S4. Multivariable Analyses of the Risk for Development of Lipid-related Diseases in Subjects with Different Dyslipidemia Phenotypes.

	Multivariable Model 1		Multivariable Model 2			Multivariable Model 1		Multivariable Model 2	
	OR/Coefficient#(95%*CI*)	*P*	*OR*/Coefficient*(95%*CI*)	*P*		*OR*/Coefficient*(95%*CI*)	*P*	*OR*/Coefficient*(95%*CI*)	*P*
**Atherosclerosis**					**Diabetes Mellitus**				
Dyslipiemia		<0.001		<0.001	Dyslipiemia		<0.001		<0.001
Type 1	1.43 (1.21,1.69)	<0.001	1.37 (1.16,1.61)	<0.001	Type 1	1.32 (1.13,1.55)	0.001	1.25 (1.06,1.47)	0.009
Type 2	1.33 (1.11,1.59)	0.002	1.27 (1.05,1.53)	0.012	Type 2	2.41 (2.06,2.81)	<0.001	1.71 (1.45,2.01)	<0.001
Type 3	0.84 (0.63,1.12)	0.227	0.84 (0.63,1.13)	0.249	Type 3	1.45 (1.15,1.84)	0.002	1.23 (0.97,1.57)	0.087
Type 4	1.54 (1.27,1.86)	<0.001	1.47 (1.20,1.80)	<0.001	Type 4	2.88 (2.43,3.41)	<0.001	1.92 (1.60,2.31)	<0.001
Type 5	0.98 (0.75,1.28)	0.861	0.97 (0.73,1.27)	0.810	Type 5	2.10 (1.70,2.60)	<0.001	1.40 (1.12,1.76)	0.004
CIMT/(1-SD TC)	0.038 (0.017,0.060)	<0.001	0.038 (0.016,0.060)	0.001	FPG/(1-SD TC)	0.132 (0.110,0.153)	<0.001	0.103 (0.082,0.124)	<0.001
CIMT/(1-SD LDL-C)	0.047 (0.025,0.068)	<0.001	0.048 (0.026,0.070)	<0.001	FPG/(1-SD LDL-C)	0.121 (0.099,0.143)	<0.001	0.084 (0.062,0.105)	<0.001
CIMT/(1-SD TG)	-0.005(-0.026,0.015)	0.621	-0.014 (-0.036,0.009)	0.242	FPG/(1-SD TG)	0.187 (0.166,0.207)	<0.001	0.148 (0.126,0.170)	<0.001
**NAFLD**					**Hyperuricemia**				
Dyslipiemia		<0.001		<0.001	Dyslipiemia		<0.001		<0.001
Type 1	1.17 (0.98,1.40)	0.090	1.10 (0.89,1.35)	0.396	Type 1	1.74 (1.46,2.08)	<0.001	1.57 (1.30,1.88)	<0.001
Type 2	3.08 (2.63,3.60)	<0.001	2.37 (1.95,2.89)	<0.001	Type 2	2.69 (2.28,3.18)	<0.001	1.69 (1.42,2.03)	<0.001
Type 3	2.68 (2.16,3.31)	<0.001	1.91 (1.48,2.47)	<0.001	Type 3	1.74 (1.37,2.20)	<0.001	1.55 (1.21,1.99)	0.001
Type 4	3.41 (2.85,4.06)	<0.001	2.44 (1.95,3.05)	<0.001	Type 4	3.04 (2.54,3.64)	<0.001	1.70 (1.39,2.08)	<0.001
Type 5	4.57 (3.73,5.59)	<0.001	3.30 (2.54,4.28)	<0.001	Type 5	3.16 (2.57,3.89)	<0.001	1.99 (1.59,2.50)	<0.001
ALT/(1-SD TG)	0.171 (0.150,0.191)	<0.001	0.144 (0.122,0.165)	<0.001	UA/(1-SD TC)	0.117 (0.097,0.136)	<0.001	0.051 (0.032,0.069)	<0.001
AST/(1-SD TG)	0.082 (0.060,0.103)	<0.001	0.084 (0.062,0.107)	<0.001	UA/(1-SD LDL-C)	0.125 (0.105,0.145)	<0.001	0.061 (0.042,0.079)	<0.001
ALT/(1-SD HDL-C)	-0.078 (-0.099,-0.056)	<0.001	-0.033 (-0.056,-0.010)	0.004	UA/(1-SD HDL-C)	-0.161(-0.180,-0.141)	<0.001	-0.092(-0.111,-0.073)	<0.001
AST/(1-SD HDL-C)	0.013 (-0.009,0.035)	0.254	0.023 (0.000,0.046)	0.050	UA/(1-SD TG)	0.192 (0.173,0.211)	<0.001	0.086 (0.067,0.104)	<0.001

Type-1, isolated hypercholesterolemia; Type-2, isolated hypertriglyceridemia; Type-3, low HDL-C level alone; Type-4,high TC/LDL-C+high TG;Type-5, high TG + low HDL-C;*OR*, odds ratio; *CI*, confidence interval; *SD*, standard deviation; CIMT, carotid intima media thickness; TC, total cholesterol; LDL-C, low density lipoprotein cholesterol; TG, triglyceridemia; HDL-C, high density lipoprotein cholesterol; FPG, fasting plasma glucose; UA,uric acids.

The multivariable model 1 was adjusted for age, sex. The multivariable model 2 was adjusted for age, sex, body mass index, smoking status, drinking status, liver function, hypertension, hyperuricemia, diabetes mellitus, NAFLD and atherosclerosis.

#Coefficients of linear correlation were estimated between lipid parameters and the value of CIMT, FPG, ALT/AST and serum uric acid.
